# Clarifying species boundaries between bocachico (*Prochilodus magdalenae)* and bocachico de Maracaibo (*Prochilodus reticulatus)* (characiformes: Prochilodontidae) using complete mitochondrial genomes

**DOI:** 10.3389/fgene.2025.1661527

**Published:** 2025-11-06

**Authors:** Jonny Yepes-Blandón, Diego Almansa-Villa, María José Benítez-Galeano, Daiana Mir, Jim Hernández-Rangel, Víctor Atencio-García, Ana Estrada-Posada, Nélida Rodríguez-Osorio

**Affiliations:** 1 GIOANE – Grupo de Investigación en Organismos Acuáticos Nativos y Exóticos, Facultad de Ciencias Exactas y Naturales, Universidad de Antioquia, Medellín, Colombia; 2 Unidad de Genómica y Bioinformática, Departamento de Ciencias Biológicas, CENUR Litoral Norte, Universidad de la República, Salto, Uruguay; 3 Laboratorio de Investigaciones Piscícolas. Facultad Experimental de Ciencias. Universidad del Zulia, Maracaibo, Venezuela; 4 FMVZ/DCA/CINPIC, Universidad de Córdoba, Montería, Colombia; 5 ISAGEN S.A. E.S.P, Medellín, Colombia

**Keywords:** Prochilodus reticulatus, Prochilodus magdalenae, phylogenetic reconstruction, time-calibrated analysis, mitochondrial

## Abstract

The accurate phylogenetic distinction between *Prochilodus magdalenae* and *Prochilodus reticulatus* (Characiformes: Prochilodontidae) has been hindered by overlapping morphology and limited sequence data. Previous studies, relying on partial mitochondrial markers, have even suggested that *Prochilodus magdalenae* and *Prochilodus reticulatus* might be a single species. This study presents three annotated complete mitochondrial genomes for *P. reticulatus* and phylogenetic analyses that contribute to resolving uncertainty around these species’ boundaries. Our phylogenetic reconstructions, using both mitochondrial markers and complete mitogenomes, consistently support the segregation of *P. magdalenae* and *P. reticulatus* into distinct clades. Bayesian time-calibrated analysis estimates their divergence at approximately 6.9 mya (10.2–4.2 mya), coinciding with the Andean Eastern Cordillera’s final uplift. This study provides essential data for future taxonomic and conservation efforts. Our findings clarify the phylogenetic relationship between these species, emphasizing the utility of complete mitogenomes and demonstrating that sequence mislabeling, probably caused by the difficulty of accurately identifying these species based on morphological characteristics, has contributed to inconsistencies in previous phylogenetic studies within the genus *Prochilodus*.

## Introduction

The genus *Prochilodus* (Characiformes: Prochilodontidae) comprises 13 species of detritivores freshwater fishes distributed throughout South American rivers in Colombia, Venezuela, French Guiana, Suriname, Brazil, Peru, Bolivia, Argentina, Paraguay, and Uruguay (Castro and Vari, 2003). However, species identification within this genus remains challenging. A recent phylogenetic study revealed that two currently recognized species, *P. rubrotaeniatus* and *P. nigricans,* seem to be composed of two genetically distinct lineages (*P. rubrotaeniatus* 1 and 2; *P. nigricans* 1 and 2) and do not form exclusive monophyletic groups. These findings prompted the authors to suggest an increase in the number of putative lineages within the genus Prochilodus from 13 to 15 (Frable et al., 2022).

Marked morphological similarities among species in the genus *Prochilodus* have been documented, making accurate species identification difficult (Castro and Vari, 2004). Despite numerous attempts to elucidate the phylogenetic relationships, taxonomical ambiguities within the genus persist. It has been pointed out that some species of the genus *Prochilodus* form single clades, while other species are composed of more than one lineage (Melo et al., 2016; Melo et al., 2018). Furthermore, the absence of complete mitochondrial genome sequences for all members of the genus, coupled with the genetic similarity observed in individual mitochondrial genes among species of the genus *Prochilodus*, adds additional challenges for species delimitation.

**FIGURE 1:**
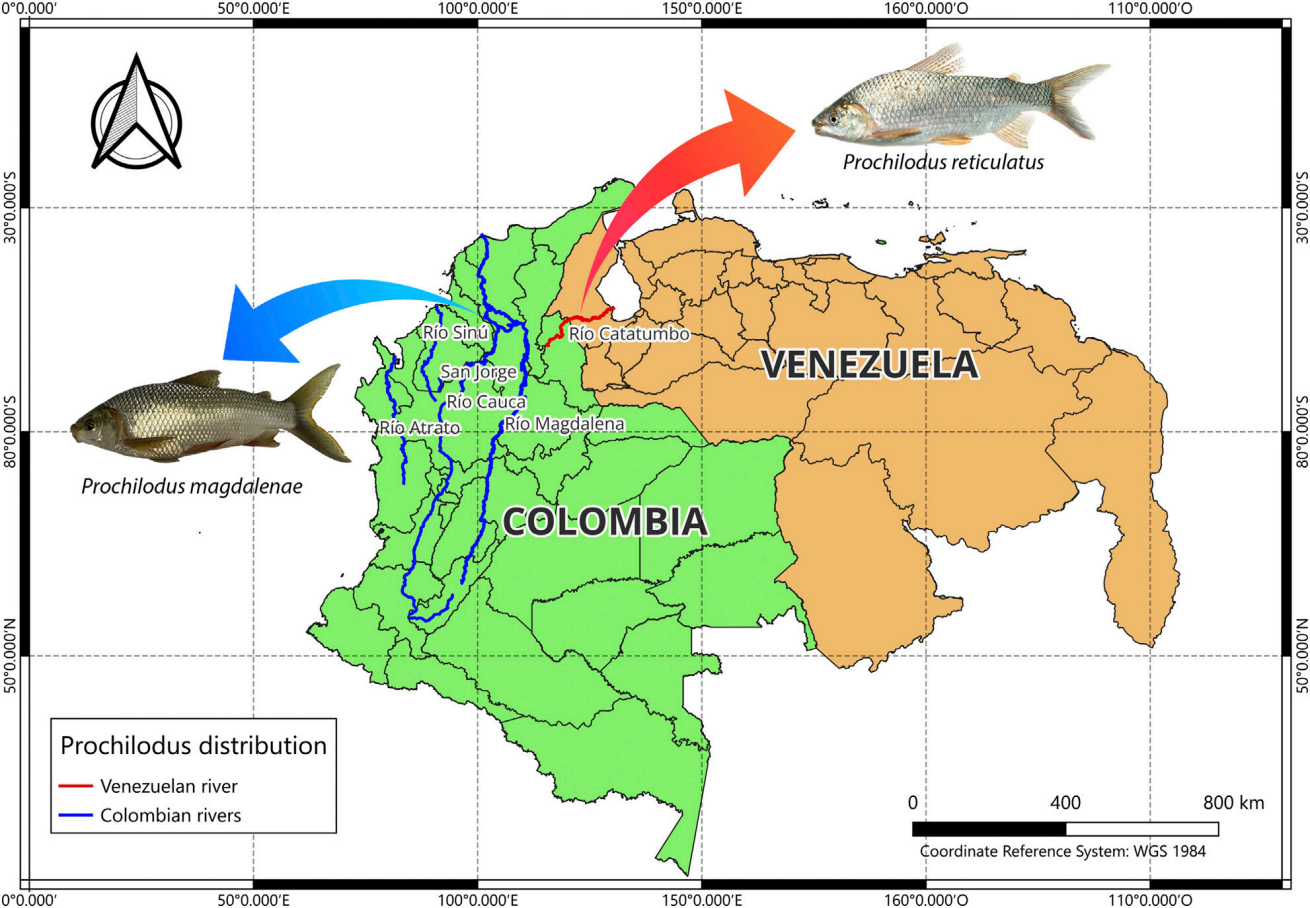
Geographic distribution of *Prochilodus magdalenae* and *P. reticulatus* in the river systems of Colombia and Venezuela. *P. magdalenae* occurs in the Magdalena, Cauca, San Jorge, Sinú, and Atrato rivers, while *P. reticulatus* inhabits the border watershed of the Catatumbo River, shared by both countries. The photograph of *P. reticulatus* was taken from Londoño-López, J. L (2023).

Five species belonging to the genus *Prochilodus* have been documented in Colombia: *P. magdalenae*, *P. reticulatus*, *P. mariae*, *P. nigricans*, and *P. rubrotaeniatus* (DoNascimiento et al., 2017). Distinction between the species *P. magdalenae* and *P. reticulatus* has primarily relied on geographical distribution, and on modal values for specific meristic characteristics (Mojica et al., 2012). *Prochilodus magdalenae* is distributed exclusively in Colombia in the Magdalena, Sinú, and Atrato rivers, at altitudes below 1,000 m above sea level, and in the Cauca River, where it can be found up to 1,500 m above sea level (DoNascimiento et al., 2017; Sidlauskas and Valderrama, 2022). In contrast, *P. reticulatus* is found in the Catatumbo River in Colombia, in mountains ranging from up to 1,000 m above sea level down to the plains where the Catatumbo River drains into Lake Maracaibo, in Venezuela (Rodríguez-Olarte et al., 2009; Mojica et al., 2012; Ortega-Lara et al., 2012) (Figure 1). A few studies report the presence of *P. reticulatus* in the Ranchería River in Colombia (Mojica et al., 2006).

At the molecular level, differentiating *P. magdalenae* and *P. reticulatus* has proven challenging. Random Amplified Polymorphic DNA (RAPD) analysis failed to distinguish between these two species (Vega-Contreras et al., 2017). Moreover, an apparent absence of differentiation observed across three mitochondrial (COI, CYTB, and 16S rRNA) and three nuclear markers (Myh6, Rag1, and Rag2), led researchers to suggest that *P. magdalenae* and *P. reticulatus* may constitute a single species, undergoing allopatric divergence (Melo et al., 2018). A recent phylogenetic study suggests that these two trans-Andean species diverged from the rest of the genus during the middle Miocene, approximately 14.4 million years ago, and that *P. reticulatus* subsequently split from *P. magdalenae* after geographic isolation in the Maracaibo system during the late Pliocene ∼2.4 million years ago (Frable et al., 2022). The current limited availability of *P. reticulatus* sequences in public genomic databases limits the possibility of conducting phylogenetic analysis for this species.

Complete mitochondrial genomes (mitogenomes) provide clear advantages over partial markers because they include the full set of protein-coding genes, rRNAs, tRNAs, and control regions. This broader coverage yields higher phylogenetic resolution and more robust evolutionary inferences (Alvarenga et al., 2024), enhances species delimitation in taxa with subtle morphological differences or recent divergence, and helps detect and correct mislabelling in public databases by enabling comparisons across multiple genomic regions.

In a prior effort, we generated a draft genome for *P. magdalenae* (GCA024036415.1), that included the assembly and annotation of its mitochondrial genome sequence (Yepes-Blandón et al., 2023). Nevertheless, the lack of a complete mitochondrial genome for *P. reticulatus*, and the limited availability of partial sequences for mitochondrial markers impeded our progress in achieving molecular phylogenetic differentiation between *P. magdalenae* and *P. reticulatus*. To address this limitation, we now present three annotated complete mitochondrial genomes of *Prochilodus reticulatus* (Characiformes: Prochilodontidae), along with a comprehensive phylogenetic analysis including two *P. magdalenae* complete mitochondrial genomes.

## Materials and methods

All animal handling procedures adhered to the guidelines outlined in the Guide for the Care and Use of Laboratory Animals (Albus 2012). Authorizations were obtained in Venezuela from the Instituto Socialista de la Pesca y Acuicultura (INSOPESCA), under permit number 1420 (5 December 2011), and in Colombia from the National Aquaculture and Fisheries Authority (AUNAP), under Resolution 0955 (27 May 2020).

### Sample collection

*Prochilodus reticulatus* samples were collected in Maracaibo, Venezuela from April to June 2020, from an area comprising four strategically chosen stations: the first station located in Congo Mirador (9° 23′37″N, 71° 48′09″W), the second in Ologá (9° 25′30″N, 71° 50′44.4″W), the third in Boca del Catatumbo (9° 21′58.80″N, 71° 42′36.61″W), and the fourth in proximity to Caño Muerto (9° 14′41″N, 71° 47′46″W). A total of 25 individuals of *P. reticulatus* were collected during the sampling process. Specimens underwent biometric assessment to record their total length, number of dorsal and anal fin rays, number of predorsal scales, and number of scales along the lateral line. Each specimen was dissected, and a sample of muscle tissue was collected, placed in a polypropylene conical tube and transported on ice to the laboratory. Muscle samples from three specimens were randomly chosen for DNA sequencing.

Additionally, to validate our previously obtained mitogenome of *Prochilodus magdalenae*, namely, Pmag_1 (Yepes-Blandón et al., 2023), a different specimen of *P. magdalenae* was collected in Santander, Colombia (7° 06′31.1″N, 73° 51′20.2″W) for DNA isolation and the generation of a second mitogenome. After sampling all specimens were discarded.

### DNA extraction and sequencing

Three different individuals of *P. reticulatus* were processed as follows: genomic DNA was extracted from muscle tissue using the GeneJET Genomic DNA purification kit (Thermo-Scientific™) following the manufacturer’s instructions and quantification of DNA was measured by absorbance on a Nano-300 (Allsheng Instruments CO., Ltd., Hangzhou, China). Sequencing libraries were prepared from 1 µg of DNA following the TruSeq Nano DNA Sample Preparation Guide (Illumina). After library preparation, 150 PE reads were generated at Macrogen (South Korea) using the Illumina Novaseq 6000 platform. The raw reads generated for *P. reticulatus* were deposited in the NCBI Sequence Read Archive (SRA) under the accession numbers SRR33454776, SRR33454777, and SRR33454778.

For the second *P. magdalenae* mitogenome (Pmag_2), high molecular weight (HMW), genomic DNA was extracted from fresh brain tissue using the MagAttract HMW DNA kit (QIAGEN) according to the manufacturer’s instructions. For library preparation, 1 µg of genomic DNA (gDNA) was used to construct four Oxford Nanopore Technologies (ONT) libraries employing the Ligation Sequencing Kit (SQK-LSK109). The DNA was first repaired and end-prepped using the NEBNext Companion Module for ONT Ligation Sequencing (NEB #E7180), following the manufacturer’s recommendations. Adapter ligation was performed using the NEBNext Module, and the libraries were purified using AMPure XP beads (Beckman Coulter) and eluted in 15 µL of elution buffer. Each library was sequenced individually on a MinION device (Oxford Nanopore Technologies) using R9.4.1 flow cells. The resulting long-read datasets were deposited in the SRA under the accession number SRR33479885.

### Read processing, mitochondrial genome assembly and annotation

Illumina raw reads from *P. reticulatus* libraries, were filtered by quality (Phred >32) and length (>100) using Trimmomatic v0.39 (Bolger et al., 2014). To ensure the accuracy and reliability of the mitochondrial genome assembly, we applied two complementary strategies using the same set of trimmed reads for each individual.

In the first strategy, reads were mapped against the *P. magdalenae* complete mitochondrial genome (Pmag_1) using Bowtie2 v2.3.0 (Langmead and Salzberg, 2012). Mapped reads were extracted with SAMtools (Danecek et al., 2021), for *de novo* mitochondrial assembly with Platanus-Allee (Kajitani et al., 2019). This approach generated guided assemblies based on a closely related mitochondrial sequence.

As a complementary approach, trimmed reads were used for whole genome *de novo* assembly using the assembly module of CLC Genomics Workbench v23.0 (QIAGEN). This approach aimed to reduce reference bias and recover potential novel variants. Among the resulting contigs, those with the highest coverage and length greater than 15,000 bp were selected and subjected to BLAST searches against complete *Prochilodus* mitochondrial genomes in NCBI, in order to identify the mitochondrial scaffold.

The assemblies obtained by both approaches were compared for cross-validation, and manual curation was performed when necessary to resolve discrepancies and ensure completeness. This process yielded one final curated mitogenome per individual, namely, Pret_1, Pret_2 and Pret_3. Annotation of the mitochondrial genomes were carried out employing Mitos2 (Donath et al., 2019), run locally on our server.

Oxford Nanopore Technologies (ONT) FAST5 files from *P. magdalenae* libraries, were basecalled with a High Accuracy model using Guppy software (ONT). Quality check, read trimming (minimum quality threshold - 7 and minimum length - 500), and quality visualization were done with MinIONQC (Lanfear et al., 2019), NanoFilt, and NanoPlot (De Coster and Rademakers, 2023), respectively. Trimmed reads were mapped to the complete mitochondrial reference genome of *P. magdalenae* (Pmag_1, GCA_024036415.1) using minimap2 (Li, 2018). The Pmag_2 mitogenome was obtained using SAMtools and BCFtools (Danecek et al., 2021) to generate a consensus sequence from the mapped reads.

### Mitochondrial gene order and codon usage analysis

To characterize the structural and compositional features of the assembled mitochondrial genomes, we extracted coding sequences based on the annotations generated by MITOS2. Codon usage was analyzed using the cusp program from the EMBOSS suite v6.6.0.0 (Rice et al., 2000), which calculates codon frequencies and relative usage within protein-coding genes.

Nucleotide composition and strand asymmetry were assessed by calculating the GC content as well as AT and GC skews, using the formulas AT skew = (A − T)/(A + T) and GC skew = (G − C)/(G + C), respectively.

Gene order was inferred from the GFF annotation and validated manually for each specimen. The strand distribution of mitochondrial genes was determined by identifying the transcriptional orientation of each feature. Genes located on the heavy (H) strand and light (L) strand were categorized accordingly.

All analyses were applied to the three mitochondrial genomes of *P. reticulatus* generated in this study (Pret_1, Pret_2, and Pret_3), as well as to the newly assembled Pmag_2 and the previously published Pmag_1 mitochondrial genome (Yepes-Blandón et al., 2023), which was re-analyzed following the same pipeline to ensure consistency.

### Phylogenetic analysis using mitochondrial markers COX1 (COI), CYTB, ATP8/ATP6, tRNA-Pro, and 16S rRNA

Phylogenetic analysis was conducted using the widely used mitochondrial marker cytochrome c oxidase subunit I gene COX1, also known as COI. All available sequences of *P. reticulatus* encompassing the same fragment of the marker were retrieved from GenBank (n = 3), together with sequences from other species belonging to *Prochilodus* and *Semaprochilodus* (n = 144). The respective sequences from Pret_1, Pret_2, Pret_3, Pmag_1, and Pmag_2 were also included. Sequence alignment was performed with MAFFT v7.307 software (Katoh and Standley, 2013), and visually examined with Aliview v1.28 (Larsson, 2014). To minimize redundancy in the data used for phylogenetic reconstruction, completely identical sequences for each species were filtered using CD-HIT (Li and Godzik, 2006).

Maximum Likelihood (ML) phylogenetic trees were inferred using IQ-TREE software v1.5.3 (Nguyen et al., 2015), employing the best-fit model of nucleotide substitution determined by the ModelFinder application (Kalyaanamoorthy et al., 2017) and 10,000 ultrafast bootstrap (UFBoot) replicates to assess branch supports (Hoang et al., 2018). Results were visualized using Figtree v1.4.4 (Rambaut, 2010) and iTOL v6 (Letunic and Bork, 2024).

Phylogenetic analyses were performed independently for each of the following mitochondrial markers: *CYTB*, *ATP8/ATP6*, *16S rRNA*, and a segment comprising the *tRNA-Pro* and *Control Region* using the same approach described for COI. These analyses aimed to compare the newly assembled mitochondrial genomes with previously reported partial sequences available in GenBank and assess phylogenetic signal across multiple loci.

Detailed information, including GenBank accession number, species name, sequence type, and genomic region for all sequences used in this study, is provided in Supplementary Table S1. Only the non-redundant sequences retained after CD-HIT filtering were used in phylogenetic reconstructions.

### Phylogenetic analysis using complete mitochondrial genomes

We conducted an alignment using MAFFT with the three curated complete *Prochilodus reticulatus* mitochondrial genome sequences, both Pmag_1 and Pmag_2 (representing *Prochilodus magdalenae*), and all available complete *Prochilodus* mitogenomes from NCBI GenBank (October 2024): *Prochilodus costatus* (KR014817.1), *Prochilodus argenteus* (NC027689.1) (Chagas et al., 2016), *Prochilodus lineatus* (KY358755.1), *Prochilodus vimboides* (NC037712.1), *Prochilodus harttii* (NC037715.1), and *Prochilodus reticulatus* (PP327417.1). Additionally, we incorporated sequences from other species belonging to the order Characiformes: *Piaractus brachypomus* (KJ993871.2) (Chen et al., 2016), *Pygocentrus nattereri* (NC015840.1), *Astyanax mexicanus* (AP011982.1) (Nakatani et al., 2011), and *Astyanax paranae* (KX609386.1) (Silva et al., 2016), as well as *Danio rerio* (NC002333.2) (Broughton et al., 2001) as outgroups.

Subsequently, ML phylogenetic analysis was conducted using IQTREE, employing the best-fit model of nucleotide substitution determined by ModelFinder and 10,000 UFBoot replicates. Results were visualized using Figtree v1.4.4 (Rambaut, 2010) and iTOL v6 (Letunic and Bork, 2024).

In addition to the analysis based on the full mitochondrial genome alignment, we performed a complementary phylogenetic analysis using a concatenated dataset of the 13 mitochondrial protein-coding genes (PCGs) extracted from the same set of mitogenomes. Each gene was aligned individually using MAFFT and concatenated into a partitioned supermatrix. Partition-specific substitution models selected by ModelFinder were as follows: TPM2u + F + G4 for *ATP6*, *COX1*, *COX2*, *NAD3*, *NAD4L*, and *NAD5*; HKY + F + I for *ATP8*; TIM2 + F + I + G4 for *CYTB*; TIM2 + F + G4 for *COX3* and *NAD1*; TPM2u + F + I + G4 for *NAD2* and *NAD4*; and HKY + F + G4 for *NAD6*. ML inference was again performed in IQ-TREE using 10,000 UFBoot replicates. This approach allowed for gene-specific modeling while maintaining consistency in taxon sampling across both analyses.

### Bayesian time-calibrated phylogeny

To estimate divergence times within *Prochilodus*, a time-calibrated phylogenetic tree was inferred using BEAST v1.10.4 software (Suchard et al., 2018). The analysis included the complete mitogenomes used in the ML analyses, except for the unverified mitogenome sequence of *P. reticulatus* (PP327417.1). The mitogenomes were partitioned into 13 segments corresponding to 13 mitochondrial protein-coding genes (PCGs). For each partition, the best-fit nucleotide substitution model was selected using the ModelFinder application. A relaxed uncorrelated lognormal molecular clock (Drummond et al., 2006) and a Birth-Death Speciation model (Gernhard, 2008) were applied.

An informative prior distribution on the root node was applied based on divergence time estimates for *Prochilodus* reported by Frable et al. (2022). This prior was derived from a fossil-calibrated Bayesian phylogeny of Prochilodontidae and was modeled using a lognormal distribution with parameters: offset = 16, mean = 4.5, and standard deviation = 5.0 in real space.

A total of six independent Markov Chain Monte Carlo (MCMC) chains were run, each for 100 million states, sampling every 1,000 states. After discarding the initial 10% of each chain as burn-in, the log and tree files were combined using LogCombiner v1.10.4 (Drummond and Rambaut, 2007) to produce a single posterior distribution. Convergence of parameters and sufficient sampling were assessed by calculating the Effective Sample Size (ESS) using Tracer v1.7.2 program (Rambaut et al., 2018). The posterior tree distribution was summarized with TreeAnnotator v1.10.4 program (Suchard et al., 2018) to generate a Maximum Clade Credibility (MCC) tree, which was visualized using the geoscalePhylo function from the strap R package (Bell and Lloyd, 2015).

## Results

### Sequencing stats, assembly and annotation

On average, Illumina sequencing yielded 192,871,097 reads for each sample of *P. reticulatus* with raw read quality exceeding Q30 for 92% of the reads. Average length after trimming was 145.3 bp. The final curated mitochondrial genome assemblies from Pret_1, Pret_2, and Pret_3 exhibited high quality and completeness, each spanning 16,696 bp, except for Pret_3, which measured 16,692 bp due to minor length variation at the control region.

**FIGURE 2:**
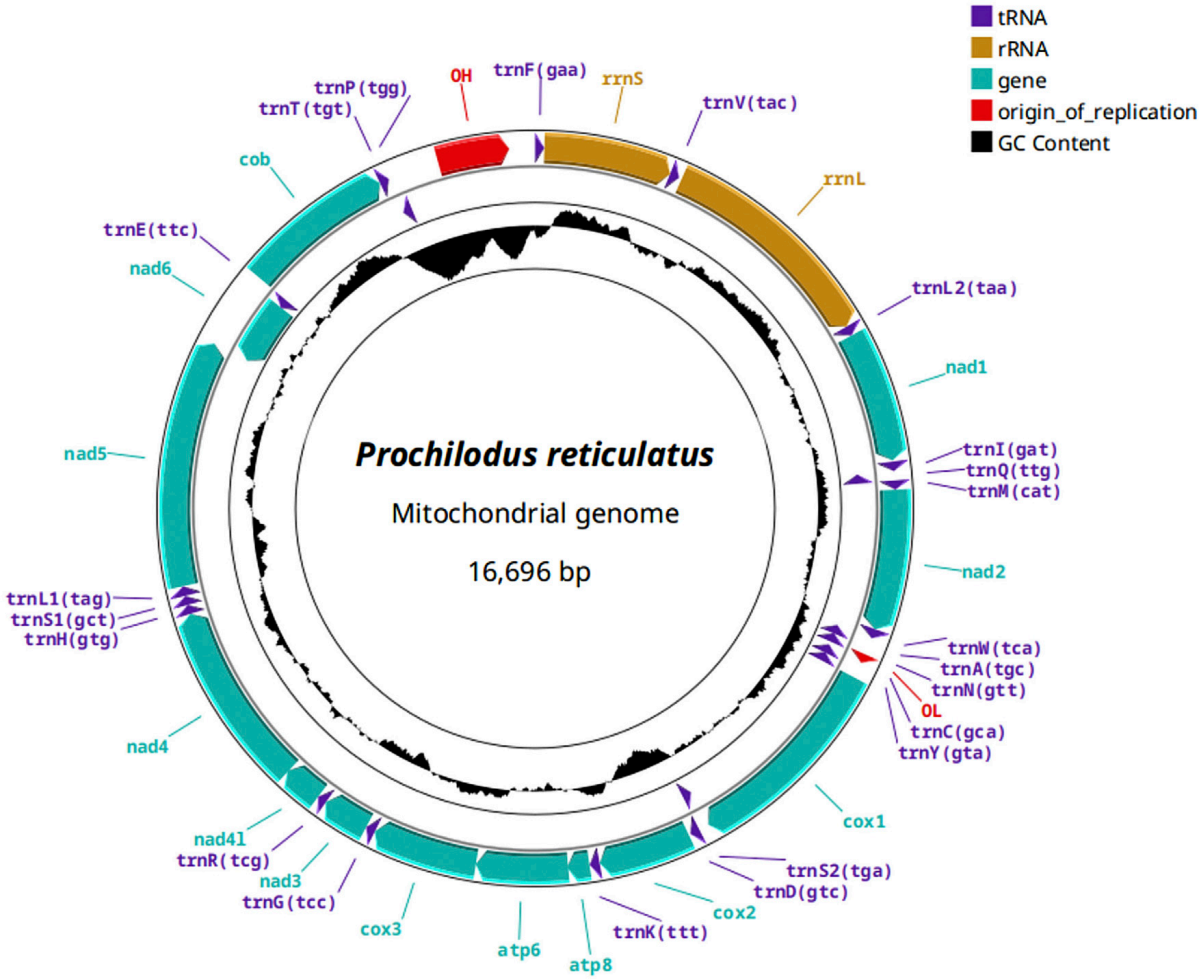
Annotation of the complete mitochondrial genome of *Prochilodus reticulatus* with protein coding genes in cyan, transfer RNAs in violet, ribosomal RNA genes in marigold color, and the control region (CR), containing the origin of replication, in red.

Annotation of the three *P. reticulatus* mitogenomes (Figure 2) showed the presence of the 13 mitochondrial protein-coding genes (CDS), 2 ribosomal RNA genes (rRNA), 22 transfer RNA genes (tRNA), and a D-loop control region, consistent with other *Prochilodus* mitogenomes. Supplementary Table S2.

Gene order was conserved across all *P. reticulatus* individuals and matched the canonical teleost mitochondrial gene arrangement. A total of 28 genes were encoded on the heavy strand (H-strand), while 9 were located on the light strand (L-strand), including *trnQ*, *trnA*, *trnN*, *trnC*, *trnY*, *trnS2*, *nad6*, *trnE*, and *trnP*. No gene rearrangements or duplications were observed. Supplementary Table S2 summarizes the accession numbers and annotation results for all mitogenomes generated in this study, plus Pmag_1.

Codon usage analysis showed that CTA (Leucine) was the most frequently used codon (53.8‰), while CGG (Arginine) was the least frequent (3.0‰). The overall GC content of protein-coding genes was 44.70%, with GC content per codon position of 51.19% (first), 41.62% (second), and 41.28% (third). These values were highly consistent across all three individuals.

For *P. magdalenae* (Pmag_2), we obtained a total of 1,262,716 ONT reads with quality scores above Q8 after trimming and filtering. The resulting mitogenome was 16,673 bp in length and exhibited the same gene content and order as the Pmag_1 sequence reported in Supplementary Table S5 of Yepes-Blandón et al. (2023). Codon usage in *P. magdalenae* was highly consistent with that observed in *P. reticulatus*, also showing a preference for CTA (Leucine, 53.7‰) and avoidance of CGG (Arginine, 3.0‰). GC content in CDS was 44.67%, with GC content by codon position of 51.46%, 41.61%, and 40.96% for the first, second, and third positions, respectively.

The AT skew and GC skew, calculated as (A − T)/(A + T) and (G−C)/(G + C), revealed consistent compositional biases across all mitogenomes: AT skew values ranged from 0.079 to 0.083 and GC skew from −0.293 to −0.297, indicating a slight preference for adenine over thymine and cytosine over guanine.

### Individual phylogenetic analysis for mitochondrial markers COX1, CYTB, 16S rRNA, ATP8/ATP6, and tRNA-Pro

**FIGURE 3:**
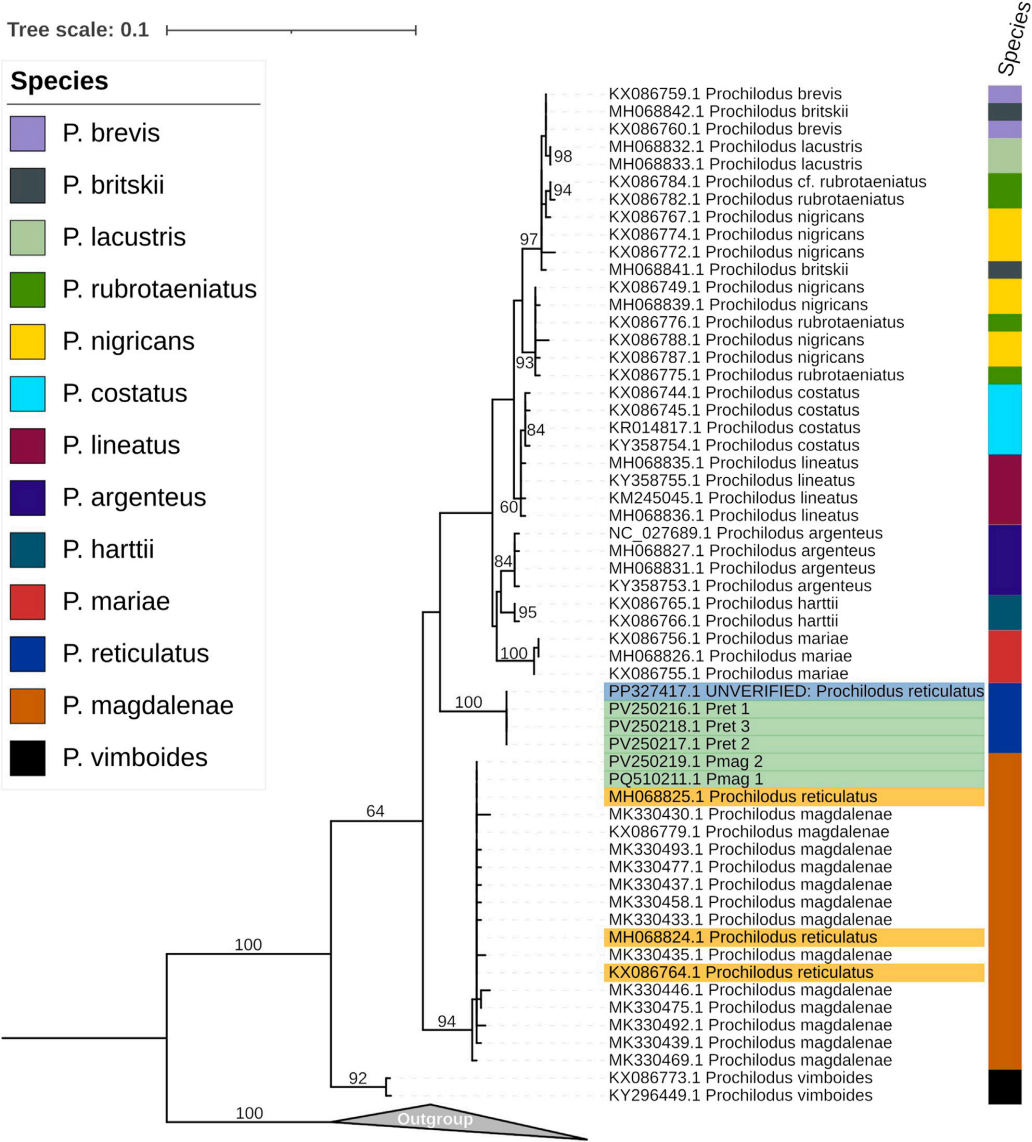
Maximum-likelihood phylogenetic tree, inferred using the K2P + G4 model, showing the relationships among *Prochilodus* species based on the COI marker alignment (587 nt). Highlighted in yellow are the GenBank accessions for *P. reticulatus* sequences that cluster within the *P. magdalenae* clade. Highlighted in green are the sequences generated in this study from *P. reticulatus* (Pret_1, Pret_2, Pret_3) and *P. magdalenae* (Pmag_2), as well as our previous *P. magdalenae* sequence (Pmag_1) Highlighted in blue is the GenBank accession for the recent *P. reticulatus* sequence that clusters within the *P. reticulatus* clade The color column on the right corresponds to previous species identifications, as indicated in the color chart. The numbers on the nodes represent ultrafast bootstrap values calculated with 10,000 bootstrap replicates. Sequences from various *Semaprochilodus* species, used as outgroup, are included in the collapsed clade.

The COI phylogenetic tree, based on a 587 bp region from the *Cox1* gene and inferred from 70 sequences, showed that three sequences of *P. reticulatus* (GenBank accession numbers MH068824, MH068825, and KX086764) are identical to all *P. magdalenae* sequences, including our Pmag_1, and Pmag_2. In contrast, our *P. reticulatus* sequences (Pret_1, Pret_2, and Pret_3), along with the corresponding COI region extracted from the unverified mitogenome sequence of *P. reticulatus* (PP327417.1), formed a single highly supported clade (UFboot = 100%) as shown in Figure 3.

**FIGURE 4:**
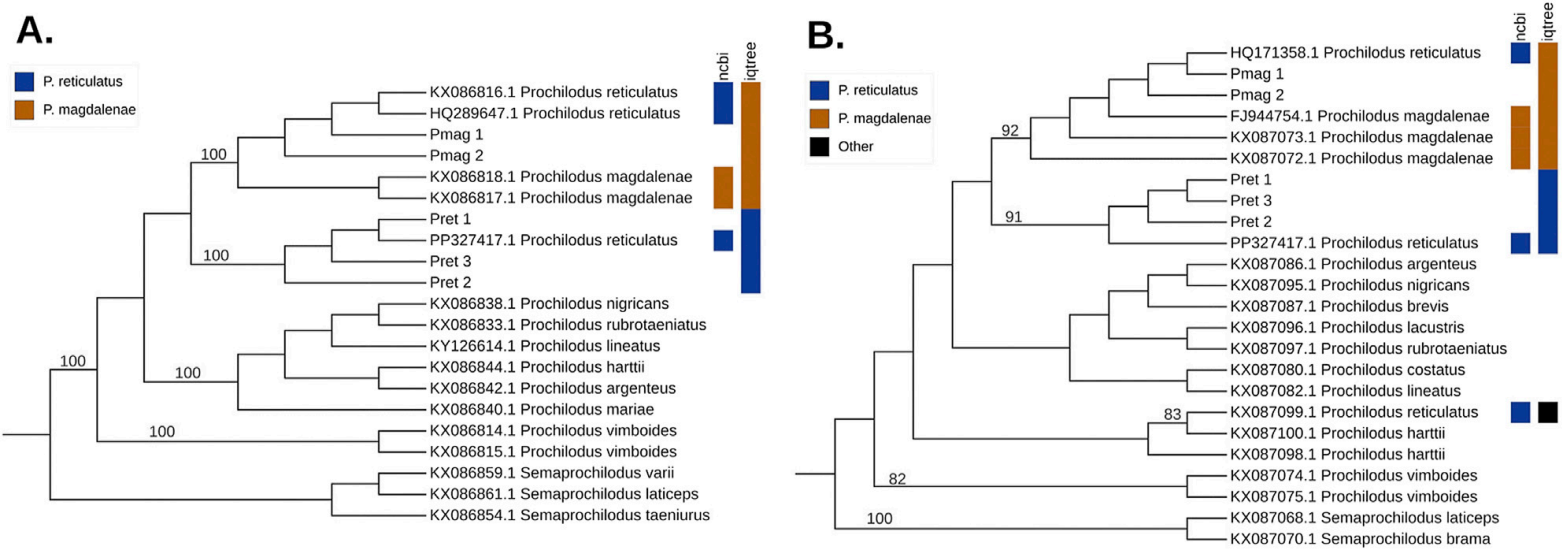
Maximum-likelihood (ML) phylogenetic trees for the mitochondrial genes **(A)** CYTB (using the K3Pu + I model), and **(B)** 16S rRNA (using the K2P + I model), with branch lengths not to scale. Numbers on the branches represent support values for well-supported nodes, calculated from 10,000 ultrafast bootstrap replicates in IQ-TREE. The colors on the right indicate taxonomic assignments from NCBI, and the rightmost column shows the species with which each sequence clusters in the tree: blue for *Prochilodus reticulatus*, tawny for *Prochilodus magdalenae*, and black for other species.

Similar results were obtained for the CYTB phylogeny (987 bp, N = 21). Only two partial *P. reticulatus* sequences were available in GenBank for this gene (KX086816 and HQ289647); the third sequence corresponds to a complete mitochondrial genome. Both partial sequences clustered within the *P. magdalenae* clade (UFboot = 100%). In contrast, our *P. reticulatus* sequences Pret_1, Pret_2, and Pret_3 together with the corresponding CYTB region from the unverified mitogenome sequences of *P. reticulatus* (PP327417.1), formed a distinct, well supported clade (UFboot = 100%) (Figure 4A).

In the 16S rRNA phylogenetic tree (594 bp, N = 24) one *P. reticulatus* sequence (HQ171358) clustered within the *P. magdalenae* clade (UFboot = 92%), while the second one (KX087099), grouped with one of the *P. harttii* sequences (UFboot = 83%). The corresponding region from the unverified mitogenome sequence of *P. reticulatus* (PP327417.1) grouped within the *P. reticulatus* clade (UFboot = 91%) (Figure 4B).

On the other hand, in the ATP8/ATP6 phylogeny, which corresponds to a region that includes segments of both ATP8 and ATP6 genes (840 bp region, N = 33) distinct clades for *P. reticulatus* and *P. magdalenae* were clearly resolved. All *P. reticulatus* ATP8/ATP6 GenBank sequences clustered with our Pret_1, Pret_2, and Pret_3 sequences, while the *P. magdalenae* GenBank sequences grouped with Pmag_1 and Pmag_2 (Supplementary Figure S1A) with UFboot = 84%. A similar pattern was observed in the phylogeny inferred from the tRNA-Pro and the control region (CR) containing the heavy strand origin of replication (1,099 bp, N = 18). Distinct and well-supported clades (UFboot = 100%) for P. *reticulatus* and *P. magdalenae* clades were obtained, with the *P. reticulatus* GenBank sequences clustering with Pret_1, Pret_2, and Pret_3 and the *P. magdalenae* GenBank sequences grouping with Pmag_1, and Pmag_2 (Supplementary Figure S1B).

### Phylogenetic analysis using complete mitochondrial genomes

**FIGURE 5:**
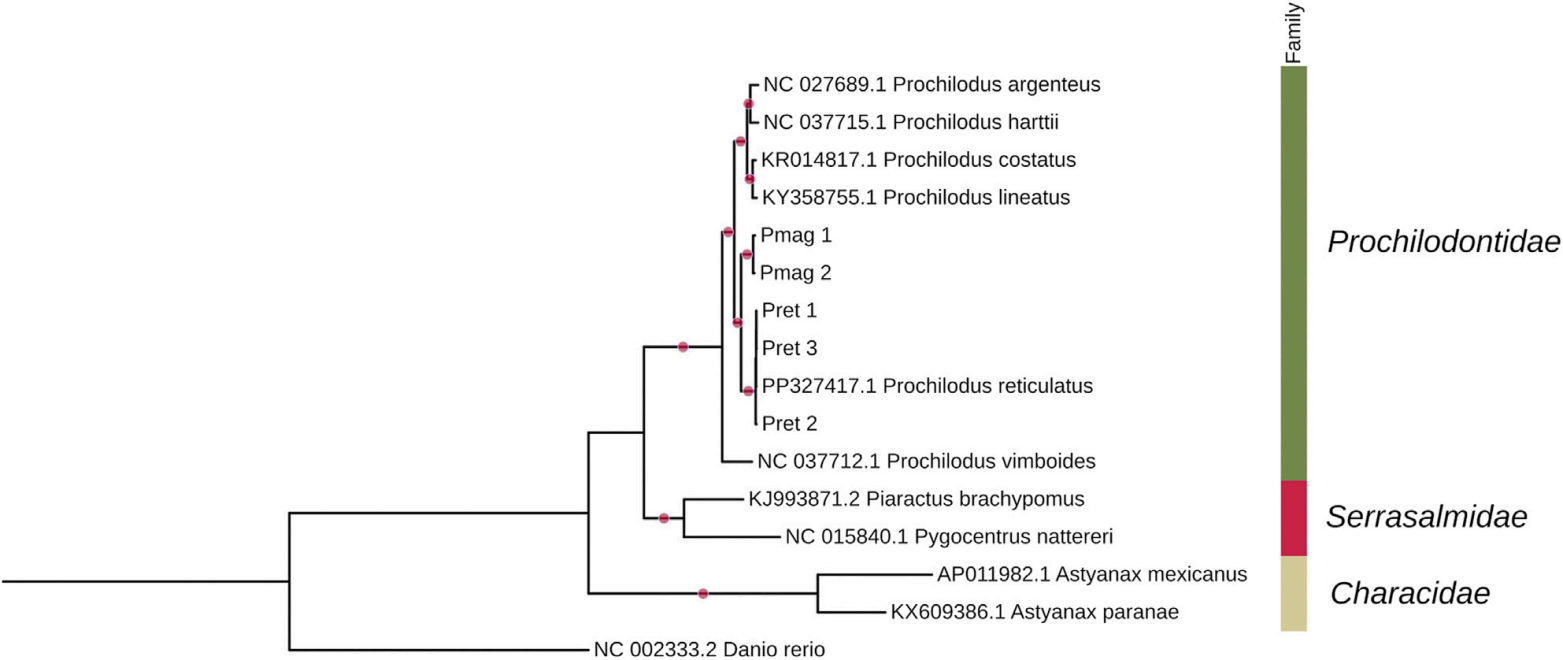
Maximum-likelihood (ML) phylogenetic tree based on a complete mitogenome sequence alignment (17,038 nucleotides) inferred using the TIM2 + I + G4 model, showing relationships among species of the genus *Prochilodus* within the Characiformes, with zebrafish as the outgroup. Bubbles on the branches represent ultrafast bootstrap values = 100%, calculated with 10,000 replicates.

A maximum likelihood (ML) phylogenetic tree was inferred using a concatenated alignment of 16 complete mitochondrial genomes (17,038 bp), representing seven *Prochilodus* species and two outgroups. The analysis resolved the relationships within Characiformes, forming well-supported clades for the genus *Prochilodus* and for each species included. Notably, *P. reticulatus* and *P. magdalenae* formed distinct, reciprocally monophyletic groups, each supported with maximal ultrafast bootstrap values (UFboot = 100%) (Figure 5).

To further explore phylogenetic resolution and assess the impact of partitioning, we performed a complementary ML analysis using a supermatrix of the 13 mitochondrial protein-coding genes (PCGs), totaling 11,477 bp, extracted from the same 16 mitogenomes. Each gene was aligned independently and treated as a separate partition. The resulting tree recovered the same overall topology as the full mitogenome analysis (Supplementary Figure S2), including identical placement of our *P. reticulatus* samples (Pret_1, Pret_2, Pret_3) and *P. magdalenae* samples (Pmag_1, Pmag_2).

Node support remained consistently high across both trees (UFboot ≥97%). The only difference observed was a slight reduction in support for the clade clustering Serrasalmidae and Prochilodontidae, which decreased from 100% in the complete mitogenome tree to 97% in the partitioned PCG-based analysis.

### Bayesian time calibrated tree

**FIGURE 6:**
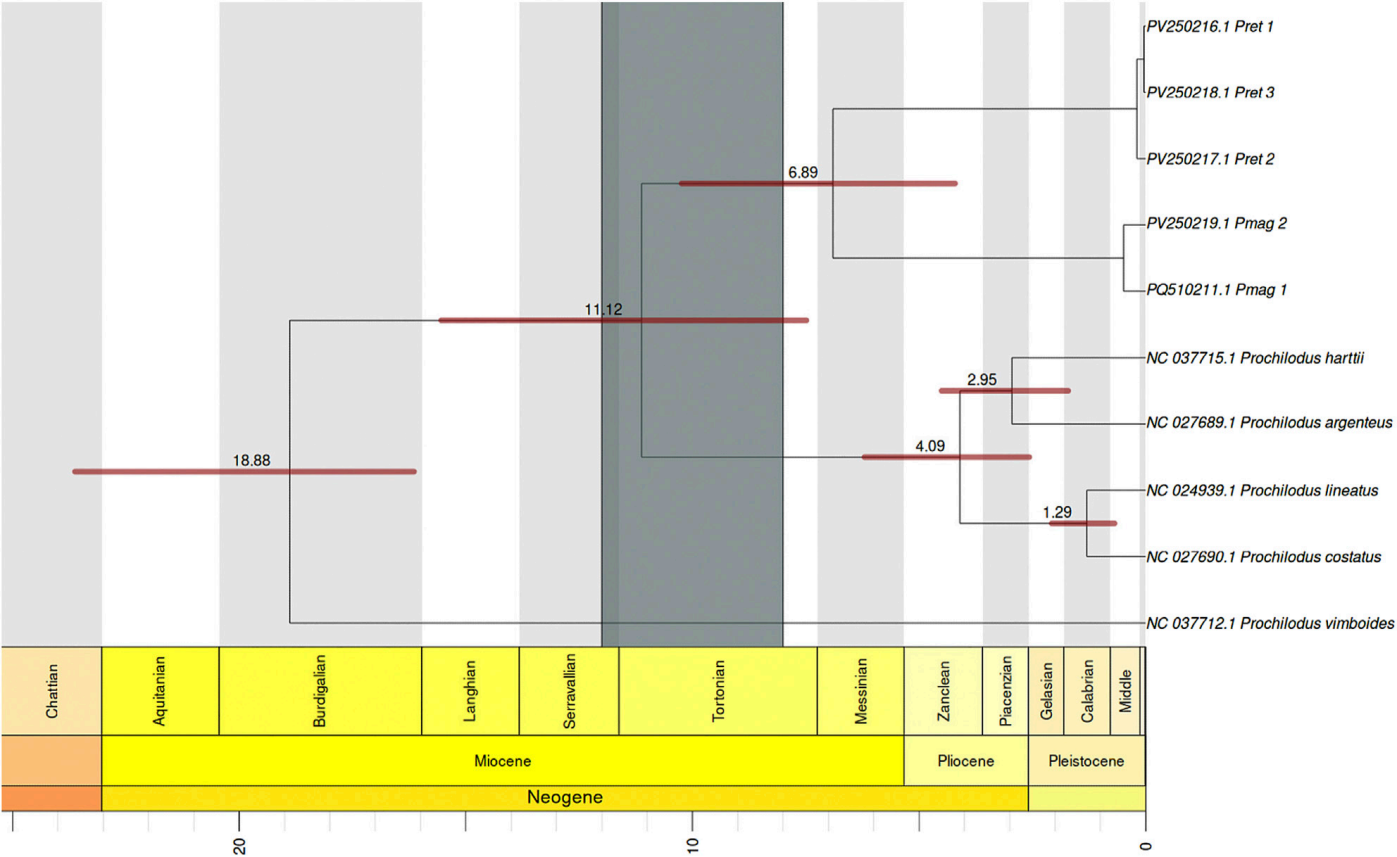
Bayesian time-calibrated phylogenetic tree inferred using BEAST illustrating the estimated divergence times within the genus Prochilodus. The numbers on the branches indicate the mean estimated age for the node in millions of years (mya), while the horizontal red bars represent the 95% HPD height. All nodes have posterior probability support values >0.95. The dark gray vertical bar over the node where P. reticulatus and P. magdalenae diverge shows the time range of the final uplift of the Eastern Andean Cordillera (12–8 mya). The light gray and white blocks behind the tree, correspond to scale periods at the bottom of the graph, which represent the Neogene and Quaternary periods with their subdivisions according to the International Chronostratigraphic Chart.

The phylogenetic reconstruction based on the 13 mitochondrial protein-coding genes (PCGs), treated as individual partitions with independent substitution models in BEAST, successfully recovered the relationships previously established with the ML analyses (Figure 6). Divergence time estimates indicated that *P. vimboides* diverged from the rest of the genus *Prochilodus* approximately 18.9 million years ago (mya) (95% HPD: 15.5–23.9 mya). Subsequently, the most recent common ancestor (MRCA) of *P. magdalenae* and *P. reticulatus* diverged from the rest of the genus around 11.1 mya (95% HPD: 7.5–15.5 mya). Within this lineage, *P. magdalenae* and *P. reticulatus* diverged from each other approximately 6.9 mya (95% HPD: 4.2–10.2 mya). Additionally, the divergence between *P. lineatus* and *P. costatus* was estimated at 1.3 mya (95% HPD: 0.7–2.1 mya), and between *P. harttii* and *P. argenteus* at 2.9 mya (95% HPD: 1.7–4.5 mya).

## Discussion

This study represents a significant advancement in resolving the taxonomic ambiguity between *P. magdalenae* and *P. reticulatus*, which has persisted despite extensive morphological and partial mitochondrial marker analyses (Melo et al., 2016; Melo et al., 2018). By analyzing several complete mitochondrial genomes for both species, we provide evidence that supports their recognition as distinct entities. Unlike previous studies that relied on limited and sometimes inconsistent molecular data (Melo et al., 2016; Melo et al., 2018), our comprehensive approach ensures a robust and validated phylogenetic framework for species identification within the genus *Prochilodus*.

Previous studies involving sequences of *P. reticulatus* and *P. magdalenae* were conducted to clarify relationships at various taxonomic levels, including the family Characidae (Oliveira et al., 2011), the family Chilodontidae (Melo et al., 2014), and the family Prochilodontidae (Melo et al., 2016). Only one study focused on the genus *Prochilodus* (Melo et al., 2018), which included several sequences for other species of this genus, but only a limited number of sequences of *P. reticulatus* and *P. magdalenae.*

The length of the mitochondrial genomes assembled in this study for both species is consistent with that of available mitogenomes for other species of the genus *Prochilodus* that range from 16,696 to 16,699 bp (do Carmo et al., 2016; Santos et al., 2021). After generating and annotating three complete mitochondrial genomes for *P. reticulatus*, we focused on studying its taxonomic relationship with *P. magdalenae,* trying to shed light on their current murky phylogenetic identification using mitochondrial markers.

Previous phylogenetic analysis, using partial COI sequences of *P. reticulatus* on GenBank (MH068824, MH068825, and KX086764) found that these were identical to those of *P. magdalenae* suggesting that this marker is not suitable for distinguishing between *P. magdalenae* and *P. reticulatus* (Melo et al., 2016). However, the same COI fragment from our three *P. reticulatus* sequences (Pret_1, Pret_2, and Pret_3), along with the corresponding region extracted from the unverified mitogenome sequence of *P. reticulatus* (PP327417.1), formed a well-supported monophyletic group (Figure 3), distinct from all other *Prochilodus* species and clearly separated from the *P. magdalenae* clade, which included the GenBank *P. reticulatus* sequences analyzed by Melo et al. (2016).

Similarly, the phylogenetic analysis of a cytochrome b (CYTB) fragment highlighted a major discrepancy with previous findings. Melo et al. (2016) reported that GenBank sequences labeled as *P. reticulatus* were identical to those of *P. magdalenae*. In contrast, our CYTB sequences (Pret_1, Pret_2, Pret_3), with the corresponding region from the unverified mitogenome sequence of *P. reticulatus* (PP327417.1), consistently formed a distinct and well-supported clade, clearly separated from *P. magdalenae* and all other species of *Prochilodus* (Figure 4A). In contrast, phylogenetic analysis of a fragment of the 16S rRNA gene yielded more confusing results, with one previously published sequence of *P. reticulatus* (HQ171358) clustering with *P. magdalenae* and another clustering with *P. harttii (*KX087099*)* (Figure 4B).

These results lead us to assume that GenBank COI (MH068824, MH068825, KX086764), CYTB (KX086816, HQ289647), and 16S rRNA (HQ171358) sequences were incorrectly labeled as *P. reticulatus* when they likely correspond to samples of *P. magdalenae*. This mislabeling may have resulted from the difficulty of accurately identifying these species based solely on morphological characteristics. Similarly, the 16S rRNA sequence KX087099, labeled as *P. reticulatus* on GenBank, appears to represent a sequence of *P. harttii*. In the original studies (Melo et al., 2016; Melo et al., 2018; Frable et al., 2022), specimens were identified based on morphological characters from museum and field collections. However, species of *Prochilodus* are often difficult to distinguish morphologically due to overlapping meristic traits, particularly between *P. reticulatus* and *P. magdalenae*. Therefore, re-examining the voucher specimens associated with these sequences would be valuable to confirm their taxonomic identity. Such misidentifications could explain the previously reported inability to reliably discriminate species within the genus *Prochilodus* using mitochondrial markers (Melo et al., 2016; Melo et al., 2018; Frable et al., 2022). The erroneous assignment of key mitochondrial sequences to *P. reticulatus,* when they actually belong to *P. magdalenae* or other species, likely contributed to the inconsistencies observed in earlier phylogenetic studies.

Overall, these findings highlight the critical issue of sequence mislabeling in public databases and its potential impact on species identification and phylogenetic inference. This issue is not isolated; species mislabelling, at different steps from sample collection to data acquisition, and the need for *in situ* surveillance protocols to prevent mislabelling have been previously discussed (Baeza, 2020). A similar pattern has been documented in other taxonomic groups, such as birds, where a sharp increase in problematic mitogenomes has been reported due to misidentification and data handling errors (Sangster and Luksenburg, 2021). Bagheri et al. (2020) identified over two million potentially misclassified proteins in the NCBI non-redundant database, attributing errors to user-submitted metadata, contamination, and computational annotation methods. Kozlov et al. (2016) developed SATIVA, a phylogeny-aware tool that detected numerous taxonomic mislabelings in public databases, emphasizing the need for automated validation methods. Furthermore, Nilsson et al. (2008) reported significant intraspecific ITS variability and misannotations within fungal sequences in international databases, underscoring the broader implications of mislabeling across diverse taxa.

Notably, the three ATP8/ATP6 sequences of *P. reticulatus* on GenBank (HQ129826, HQ129827, HQ129828) formed a monophyletic group with the corresponding region from an unverified mitogenome of *P. reticulatus* (PP327417.1), and Pret_1, Pret_2, and Pret_3 sequences. The same was observed for the long tRNA-Pro and control region (CR) sequences of *P. reticulatus* on GenBank (HQ129477, HQ129478, and HQ129479). These six sequences were submitted to GenBank as part of an unpublished study based on molecular systematics and biogeography of a South American characiform, which might explain why they are not referred to in the scientific literature. The topology of the trees for ATP8/ATP6 and tRNA-Pro and CR (Supplementary Figure S1A, B) validates our mitogenome sequences, confirms the clear distinction between both species and supports the use of these markers for DNA-based identification of *P. magdalenae* and *P. reticulatus* identification.

Complete mitochondrial genome analyses revealed that *P. magdalenae* and *P. reticulatus* form well-supported clades (Figure 5), which supports the value of species identification using complete mitogenomes. Similar advantages have been reported in other groups. For example, in *Barilius malabaricus* (Cyprinidae), complete mitogenome sequencing provided better phylogenetic resolution and more accurate species delimitation than single-gene analyses (Prabhu et al., 2020). Likewise, in the sea star genus *Henricia*, mitogenome-based phylogenies yielded clearer interspecific relationships than those inferred from partial mitochondrial markers (Alboasud et al., 2024). In fungal species such as *Phellinus igniarius*, complete mitochondrial genome analysis also offered improved insights into phylogenetic relationships compared to fragment-based approaches (He et al., 2024).

Our ML tree based on the full mitogenome recovered a topology consistent with previous descriptions of the genus (Melo et al., 2018; Frable et al., 2022), but with considerable sequence divergence and high support values (UFboot = 100%) for the *P. reticulatus* and *P. magdalenae* clades. A complementary ML analysis using a concatenated alignment of the 13 mitochondrial protein-coding genes (PCGs) produced the same overall topology, with only a minor reduction in support for the clade uniting Serrasalmidae and Prochilodontidae (UFboot = 97%). This congruence suggests that, for ML-based phylogenetic inference, both approaches—using the complete mitochondrion or the 13 PCGs—are equally effective in recovering robust and consistent relationships among taxa.

Similarly, the dated phylogenetic reconstruction recovered distinct and well-supported clades for both species (posterior probability >95%), confirming their reciprocal monophyly and long-term evolutionary separation. The divergence between *P. magdalenae* and *P. reticulatus* was estimated at 6.9 mya (10.2–4.2 mya), coinciding with the final uplift of the Andean Eastern Cordillera, as previously reported (Garzione et al., 2008; Schaefer, 2011). This finding is consistent with reports for other species in the region, belonging to the genus *Pseudoplatystoma* (Torrico et al., 2009) and the genus *Ichthyoelephas*, another member of the family Prochilodontidae (Frable et al., 2022), which also underwent allopatric speciation due to the final uplift of the Andes Mountain and the separation of the Magdalena basin from other basins around 10 mya.

Contrastingly, Frable et al. (2022) estimated a more recent divergence time of 2.4 mya for *P. reticulatus* and *P. magdalenae*, hypothesizing geographic isolation within the Lake Maracaibo system. We suggest that the inclusion of mislabeled *P. reticulatus* sequences in their datasets, as identified in our mitochondrial gene ML analyses, hindered accurate phylogenetic inference and node dating. Specifically, while some sequences labeled as *P. reticulatus* corresponded to true *P. reticulatus* individuals, others were actually derived from *P. magdalenae*. Consequently, comparisons involved *P. magdalenae* sequences against a mosaic of genuine *P. reticulatus* and misidentified *P. magdalenae* sequences. This mixture reduced the observed genetic divergence between species and likely resulted in an underestimation of the divergence time. Nevertheless, our divergence time estimations for other species within the genus *Prochilodus* were consistent with those reported by Frable et al. (2022).

## Conclusion

This study successfully resolves the long-standing taxonomic ambiguity between *P. magdalenae* and *P*. *reticulatus*. By generating complete mitochondrial genomes for both species and conducting different phylogenetic analyses, we provide compelling evidence supporting their recognition as distinct species. Although previous studies using mitochondrial markers, such as COI and CYTB, failed to differentiate between these species, our results indicate that this failure was likely due to wrongful species or sequence identification. When correctly identified sequences of *P. reticulatus* are employed, these markers can reliably distinguish *P. reticulatus* from *P. magdalenae*. Our findings highlight the limitations of relying solely on partial mitochondrial markers and suggest that sequence mislabeling has contributed to inconsistencies in previous phylogenetic studies. We emphasize the importance of using vouchered specimens, deposited in recognized collections with traceable metadata, to ensure the reliability of species identification in molecular studies.

The estimated divergence time of approximately 7 million years between *P. magdalenae* and *P. reticulatus* aligns with known geological events that explain their divergence. This research not only clarifies the taxonomic status of both species but also provides valuable insights into the evolutionary history of the genus *Prochilodus*. These findings have significant implications for future studies of the biogeography, conservation, and fisheries management strategies of these ecologically and economically important fish species. Additionally, our study demonstrates the utility of long-read sequencing using ONT portable sequencer, as an in-house alternative for generating complete mitogenomes with sufficient accuracy for phylogenetic purposes. Our validated mitochondrial genomes and comprehensive phylogenetic framework offer a reliable tool for authorities and researchers, facilitating precise monitoring and management of these ecologically and economically important species in Colombia and Venezuela.

## Data Availability

The complete mitochondrial genome sequences generated in this study have been deposited in NCBI GenBank under the following accession numbers: Pret_1 (PV250216), Pret_2 (PV250217), Pret_3 (PV250218), and Pmag_2 (PV250219). The corresponding raw sequencing data have been submitted to the NCBI Sequence Read Archive (SRA) under the following accession codes: SRR33454776, SRR33454777, and SRR33454778 for P. reticulatus samples, and SRR33479885 for P. magdalenae.

## Supplementary material

SUPPLEMENTARY FIGURE 1 Maximum-likelihood (ML) phylogenetic trees for the mitochondrial genes **(A)** ATP8/ATP6, and **(B)** tRNA-Pro, both using the HKY + G4 model, with branch lengths not to scale. Numbers on the branches represent support values for well-supported nodes, calculated from 10,000 ultrafast bootstrap replicates in IQ-TREE. The colors on the right indicate taxonomic assignments from NCBI, and the rightmost column shows the species with which each sequence clusters in the tree: blue for *Prochilodus reticulatus*, and tawny for *Prochilodus magdalenae*.

SUPPLEMENTARY FIGURE 2 Side-by-side comparison of maximum likelihood (ML) phylogenies inferred using (left, 17039 positions) the complete mitochondrial genome as a single partition and (right, 11477 positions) the 13 mitochondrial protein-coding genes (PCGs) treated as separate partitions with independent substitution models. Both analyses were based on the same set of mitogenomes and performed under the best-fit models selected using ModelFinder. The partitioned PCG approach accounted for differences in substitution patterns among genes and codon positions. Phylogenetic inference was conducted with 10,000 ultrafast bootstrap replicates in both cases. Topologies were identical between the two trees, with uniformly high node support (UFboot ≥97%). Red circles mark nodes with bootstrap values of 100. The only difference observed was a slight reduction in support for the Serrasalmidae + Prochilodontidae clade in the partitioned tree (97%) compared to the complete genome tree (100%), indicating the overall robustness of the phylogenetic inference despite methodological differences.

## References

[B1] Alboasud M. Jeong H. Lee T. (2024). Complete mitochondrial genomes and phylogenetic analysis of genus henricia (asteroidea: spinulosida: echinasteridae). Int. J. Mol. Sci. 25 (11), 5575. 10.3390/ijms25115575 38891763 PMC11171911

[B2] Alvarenga M. D’Elia A. K. P. Rocha G. Arantes C. A. Henning F. De Vasconcelos A. T. R. (2024). Mitochondrial genome structure and composition in 70 fishes: a key resource for fisheries management in the South Atlantic. BMC Genomics 25 (1), 215. 10.1186/s12864-024-10035-5 38413941 PMC10898094

[B3] Baeza J. A. (2020). Yes, we can use it: a formal test on the accuracy of low-pass nanopore long-read sequencing for mitophylogenomics and barcoding research using the Caribbean spiny lobster Panulirus argus. BMC Genomics 21 (1), 882. 10.1186/s12864-020-07292-5 33297960 PMC7726883

[B4] Bagheri H. Severin A. J. Rajan H. (2020). Detecting and correcting misclassified sequences in the large-scale public databases. Bioinformatics 36 (18), 4699–4705. 10.1093/bioinformatics/btaa586 32579213 PMC7821992

[B5] Bell M. A. Lloyd G. T. (2015). Strap: an R package for plotting phylogenies against stratigraphy and assessing their stratigraphic congruence. Palaeontology 58 (2), 379–389. 10.1111/pala.12142

[B6] Bolger A. M. Lohse M. Usadel B. (2014). Trimmomatic: a flexible trimmer for Illumina sequence data. Bioinformatics 30 (15), 2114–2120. 10.1093/bioinformatics/btu170 24695404 PMC4103590

[B7] Broughton R. E. Milam J. E. Roe B. A. (2001). The complete sequence of the zebrafish (Danio rerio) mitochondrial genome and evolutionary patterns in vertebrate mitochondrial DNA. Genome Res. 11 (11), 1958–1967. 10.1101/gr.156801 11691861 PMC311132

[B8] Castro R. M. C. Vari R. P. (2003). “Prochilodontidae (Fannel mouth characiforms),” in Checklist of the freshwater fishes of south and central America. Editors Reis R. E. Kullander S. O. Ferraris Jr. C. J. (Porto Alegre, Brasil), 65–70.

[B9] Castro R. M. C. Vari R. P. (2004). Detritivores of the South American fish family Prochilodontidae (Teleostei:Ostariophysi:Characiformes): a phylogenetic and revisionary study. Smithson. Contrib. Zool., 1–189. 10.5479/si.00810282.622

[B10] Chagas A. T. de A. Carmo A. O. Costa M. A. Resende L. C. Brandão Dias P. F. P. Martins A. P. V. (2016). Description and comparison of two economically important fish species mitogenomes: prochilodus argenteus and Prochilodus costatus (Characiformes, Prochilodontidae). Mitochondrial DNA A DNA Mapp. Seq. Anal. 27 (4), 2852–2853. 10.3109/19401736.2015.1053125 26171874

[B11] Chen H. Li S. Xie Z. Zhang Y. Zhu C. Deng S. (2016). The complete mitochondrial genome of the Piaractus brachypomus (Characiformes: characidae). Anal 27 (2), 1289–1290. 10.3109/19401736.2014.945560 25090392

[B12] Danecek P. Bonfield J. K. Liddle J. Marshall J. Ohan V. Pollard M. O. (2021). Twelve years of SAMtools and BCFtools. GigaScience 10 (2), giab008. 10.1093/gigascience/giab008 33590861 PMC7931819

[B13] De Coster W. Rademakers R. (2023). “NanoPack2: population-scale evaluation of long-read sequencing data,” Bioinformatics, 39. 10.1093/bioinformatics/btad311 37171891 PMC10196664

[B14] do Carmo A. O. Brandão D. Ferreira Pinto P. Martins A. Vimieiro P. Alessandra G. (2016). Complete mitochondrial genome sequence of Prochilodus lineatus (Characiformes, Prochilodontidae). Mitochondrial DNA Part A 27 (3), 1946–1947. 10.3109/19401736.2014.971300 25329266

[B15] DoNascimiento C. Herrera-Collazos E. E. Herrera-R G. A. Ortega-Lara A. Villa-Navarro F. A. Oviedo J. S. U. (2017). Checklist of the freshwater fishes of Colombia: a Darwin Core alternative to the updating problem. Zookeys 708, 25–138. 10.3897/zookeys.708.13897 29118633 PMC5674168

[B16] Donath A. Jühling F. Al-Arab M. Bernhart S. H. Reinhardt F. Stadler P. F. (2019). Improved annotation of protein-coding genes boundaries in metazoan mitochondrial genomes. Nucleic Acids Res. 47 (20), 10543–10552. 10.1093/nar/gkz833 31584075 PMC6847864

[B17] Drummond A. J. Rambaut A. (2007). BEAST: bayesian evolutionary analysis by sampling trees. BMC Evol. Biol. 7 (1), 214. 10.1186/1471-2148-7-214 17996036 PMC2247476

[B18] Drummond A. J. Ho S. Y. W. Phillips M. J. Rambaut A. (2006). Relaxed phylogenetics and dating with confidence. PLOS Biol. 4 (5), e88. 10.1371/journal.pbio.0040088 16683862 PMC1395354

[B19] Frable B. W. Melo B. F. Fontenelle J. P. Oliveira C. Sidlauskas B. L. (2022). Biogeographic reconstruction of the migratory Neotropical fish family Prochilodontidae (Teleostei: characiformes). Zool. Scr. 51 (3), 348–364. 10.1111/zsc.12531

[B20] Garzione C. N. Hoke G. D. Libarkin J. C. Withers S. MacFadden B. Eiler J. (2008). Rise of the andes. Science 320 (5881), 1304–1307. 10.1126/science.1148615 18535236

[B21] Gernhard T. (2008). The conditioned reconstructed process. J. Theor. Biol. 253 (4), 769–778. 10.1016/j.jtbi.2008.04.005 18538793

[B22] He Q. Jiang Y. Li Y. Guan T. Jing X. Meng C. (2024). Complete mitochondrial genome sequencing and phylogenetic analysis of Phellinus igniarius. Sci. Rep. 14 (1), 31109. 10.1038/s41598-024-82372-0 39732982 PMC11682317

[B23] Hoang D. T. Chernomor O. Von Haeseler A. Minh B. Q. Vinh L. S. (2018). UFBoot2: improving the ultrafast bootstrap approximation. Mol. Biol. Evol. 35 (2), 518–522. 10.1093/molbev/msx281 29077904 PMC5850222

[B24] Kajitani R. Yoshimura D. Okuno M. Minakuchi Y. Kagoshima H. Fujiyama A. (2019). Platanus-allee is a *de novo* haplotype assembler enabling a comprehensive access to divergent heterozygous regions. Nat. Commun. 10 (1), 1702. 10.1038/s41467-019-09575-2 30979905 PMC6461651

[B25] Kalyaanamoorthy S. Minh B. Q. Wong T. K. F. Von Haeseler A. Jermiin L. S. (2017). ModelFinder: fast model selection for accurate phylogenetic estimates. Nat. Methods 14 (6), 587–589. 10.1038/nmeth.4285 28481363 PMC5453245

[B26] Katoh K. Standley D. M. (2013). MAFFT multiple sequence alignment software version 7: improvements in performance and usability. Mol. Biol. Evol. 30 (4), 772–780. 10.1093/molbev/mst010 23329690 PMC3603318

[B27] Kozlov A. M. Zhang J. Yilmaz P. Glöckner F. O. Stamatakis A. (2016). Phylogeny-aware identification and correction of taxonomically mislabeled sequences. Nucleic Acids Res. 44 (11), 5022–5033. 10.1093/nar/gkw396 27166378 PMC4914121

[B28] Lanfear R. Schalamun M. Kainer D. Wang W. Schwessinger B. (2019). MinIONQC: fast and simple quality control for MinION sequencing data. Bioinformatics 35 (3), 523–525. 10.1093/bioinformatics/bty654 30052755 PMC6361240

[B29] Langmead B. Salzberg S. L. (2012). Fast gapped-read alignment with Bowtie 2. Nat. Methods 9 (4), 357–359. 10.1038/nmeth.1923 22388286 PMC3322381

[B30] Larsson A. (2014). AliView: a fast and lightweight alignment viewer and editor for large datasets. Bioinformatics 30 (22), 3276–3278. 10.1093/bioinformatics/btu531 25095880 PMC4221126

[B31] Letunic I. Bork P. (2024). Interactive Tree of Life (iTOL) v6: recent updates to the phylogenetic tree display and annotation tool. Nucleic Acids Res. 52 (W1), W78–W82. 10.1093/nar/gkae268 38613393 PMC11223838

[B32] Li H. (2018). Minimap2: pairwise alignment for nucleotide sequences. Bioinformatics 34 (18), 3094–3100. 10.1093/bioinformatics/bty191 29750242 PMC6137996

[B33] Li W. Godzik A. (2006). Cd-hit: a fast program for clustering and comparing large sets of protein or nucleotide sequences. Bioinformatics 22 (13), 1658–1659. 10.1093/bioinformatics/btl158 16731699

[B34] Melo B. F. Sidlauskas B. L. Hoekzema K. Vari R. P. Oliveira C. (2014). The first molecular phylogeny of Chilodontidae (Teleostei: ostariophysi: characiformes) reveals cryptic biodiversity and taxonomic uncertainty. Mol. Phylogenetics Evol. 70, 286–295. 10.1016/j.ympev.2013.09.025 24120449

[B35] Melo B. F. Sidlauskas B. L. Hoekzema K. Frable B. W. Vari R. P. Oliveira C. (2016). Molecular phylogenetics of the Neotropical fish family Prochilodontidae (Teleostei: characiformes). Mol. Phylogenetics Evol. 102, 189–201. 10.1016/j.ympev.2016.05.037 27262428

[B36] Melo B. F. Dorini B. F. Foresti F. Oliveira C. (2018). Little divergence among mitochondrial lineages of Prochilodus (Teleostei, Characiformes). Front. Genet. 9, 107. 10.3389/fgene.2018.00107 29670644 PMC5893770

[B37] Mojica J. I. Castellanos C. Sánchez-Duarte P. Díaz C. (2006). Peces de la cuenca del río Ranchería, La Guajira, Colombia. Biota Colomb. 7 (1), 129–142. Available online at: https://revistas.humboldt.org.co/index.php/biota/article/view/168.

[B38] Mojica J. I. Acero A. Acosta-Santos A. A. Agudelo-Córdoba E. Agudelo-Zamora H. D. Ajiaco-Martínez R. E. (2012). Libro rojo de peces dulceacuícolas de Colombia (2012). Bogotá, Colombia: Instituto de Investigación de Recursos Biológicos Alexander von Humboldt. Available online at: http://hdl.handle.net/20.500.11761/34197.

[B39] Nakatani M. Miya M. Mabuchi K. Saitoh K. Nishida M. (2011). Evolutionary history of Otophysi (Teleostei), a major clade of the modern freshwater fishes: pangaean origin and Mesozoic radiation. BMC Evol. Biol. 11, 177. 10.1186/1471-2148-11-177 21693066 PMC3141434

[B40] Nguyen L.-T. Schmidt H. A. Von Haeseler A. Minh B. Q. (2015). IQ-TREE: a fast and effective stochastic Algorithm for estimating maximum-likelihood phylogenies. Mol. Biol. Evol. 32 (1), 268–274. 10.1093/molbev/msu300 25371430 PMC4271533

[B41] Nilsson R. H. Kristiansson E. Ryberg M. Hallenberg N. Larsson K.-H. (2008). Intraspecific ITS variability in the kingdom fungi as expressed in the international sequence databases and its implications for molecular species identification. Evol. Bioinform Online. 4, 193–201. 10.4137/ebo.s653 19204817 PMC2614188

[B42] Oliveira C. Avelino G. S. Abe K. T. Mariguela T. C. Benine R. C. Ortí G. (2011). Phylogenetic relationships within the speciose family Characidae (teleostei: ostariophysi: characiformes) based on multilocus analysis and extensive ingroup sampling. BMC Evol. Biol. 11 (1), 275. 10.1186/1471-2148-11-275 21943181 PMC3190395

[B43] Ortega-Lara A. Lasso-Alcalá O. M. Lasso C. A. Andrade De P. G. Bogotá-Gregory J. D. (2012). Peces de la subcuenca del río Catatumbo, cuenca del Lago de Maracaibo, Colombia y Venezuela. 10.21068/bc.v13i1.258

[B44] Prabhu V. R. Singha H. S. Kumar R. G. Gopalakrishnan A. Nagarajan M. (2020). Characterization of the complete mitochondrial genome of *Barilius malabaricus* and its phylogenetic implications. Genomics 112 (3), 2154–2163. 10.1016/j.ygeno.2019.12.009 31843505

[B45] Rambaut A. (2010). FigTree [Computer software]. University of Edinburgh, Institute of Evolutionary Biology. Available online at: https://tree.bio.ed.ac.uk/software/figtree/ .

[B46] Rambaut A. Drummond A. J. Xie D. Baele G. Suchard M. A. (2018). Posterior summarization in Bayesian phylogenetics using tracer 1.7. Syst. Biol. 67 (5), 901–904. 10.1093/sysbio/syy032 29718447 PMC6101584

[B47] Rice P. Longden I. Bleasby A. (2000). EMBOSS: the European molecular biology open software suite. Trends Genet. 16 (6), 276–277. 10.1016/S0168-9525(00)02024-2 10827456

[B48] Rodríguez-Olarte D. Taphorn D. C. Lobón-Cerviá J. (2009). Patterns of freshwater fishes of the Caribbean versant of Venezuela. Int. Rev. Hydrobiology 94 (1), 67–90. 10.1002/iroh.200711070

[B49] Sangster G. Luksenburg J. A. (2021). Sharp increase of problematic mitogenomes of birds: causes, consequences, and remedies. Genome Biol. Evol. 13 (9), evab210. 10.1093/gbe/evab210 34505894 PMC8462277

[B50] Santos R. P. Melo B. F. Yazbeck G. M. Oliveira R. S. Hilário H. O. Prosdocimi F. (2021). Diversification of prochilodus in the eastern Brazilian shield: evidence from complete mitochondrial genomes (teleostei, prochilodontidae). J. Zoological Syst. Evol. Res. 59 (5), 1053–1063. 10.1111/jzs.12475

[B51] Schaefer S. (2011). “The andes: riding the tectonic uplift,” in Historical biogeography of neotropical freshwater fishes. Editor Albert J. (Oakland, CA), 259–278. 10.1525/california/9780520268685.003.0016

[B52] Sidlauskas B. L. Valderrama M. (2022). IUCN red list of threatened species: Prochilodus magdalenae. The IUCN Red List Threat. Species. Available online at: https://www.iucnredlist.org/en.

[B53] Silva D. M. Z. de A. Utsunomia R. Ruiz-Ruano F. J. Oliveira C. Foresti F. (2016). The complete mitochondrial genome sequence of astyanax paranae(Teleostei: characiformes). Mitochondrial DNA B Resour. 1 (1), 586–587. 10.1080/23802359.2016.1222251 33490410 PMC7800300

[B54] Suchard M. A. Lemey P. Baele G. Ayres D. L. Drummond A. J. Rambaut A. (2018). Bayesian phylogenetic and phylodynamic data integration using BEAST 1.10. Virus Evol. 4 (1), vey016. 10.1093/ve/vey016 29942656 PMC6007674

[B55] Torrico J. P. Hubert N. Desmarais E. Duponchelle F. Nuñez Rodriguez J. Montoya-Burgos J. (2009). Molecular phylogeny of the genus *pseudoplatystoma* (bleeker, 1862): biogeographic and evolutionary implications. Mol. Phylogenetics Evol. 51 (3), 588–594. 10.1016/j.ympev.2008.11.019 19070672

[B56] Vega-Contreras N. A. Galvis Serrano N. F. Salazar Mercado S. A. (2017). Relaciones evolutivas de los peces Prochilodus reticulatus y Prochilodus magdalenae (Characiformes: prochilodontidae). Rev. Ciencias 21 (1), 161–173. 10.25100/rc.v21i1.6348

[B57] Yepes-Blandón J. A. Bian C. Benítez-Galeano M. J. Aristizabal-Regino J. L. Estrada-Posada A. L. Mir D. (2023). Draft genome assembly for the Colombian freshwater bocachico fish, Prochilodus magdalenae. Front. Genet. 13, 989788. 10.3389/fgene.2022.989788 36744175 PMC9893009

