# Supplementary figures and images for: Clarifying species boundaries between bocachico (Prochilodus magdalenae) and bocachico de Maracaibo (Prochilodus reticulatus) (characiformes: Prochilodontidae) using complete mitochondrial genomes
Source: Front Genet. 2025 Nov 6;16:1661527. doi: 10.3389/fgene.2025.1661527 (PMC12631445; doi:10.3389/fgene.2025.1661527)

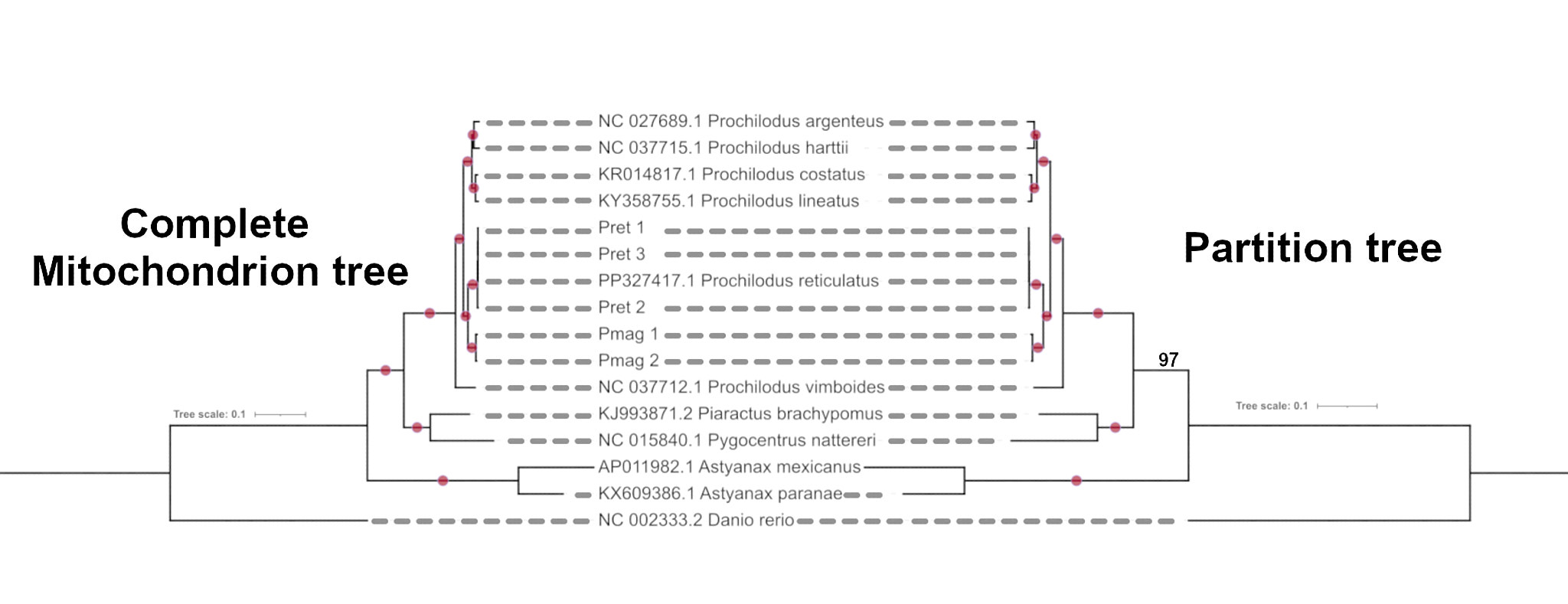

Supplement: Supplementary file 2 [file Image3.jpeg]

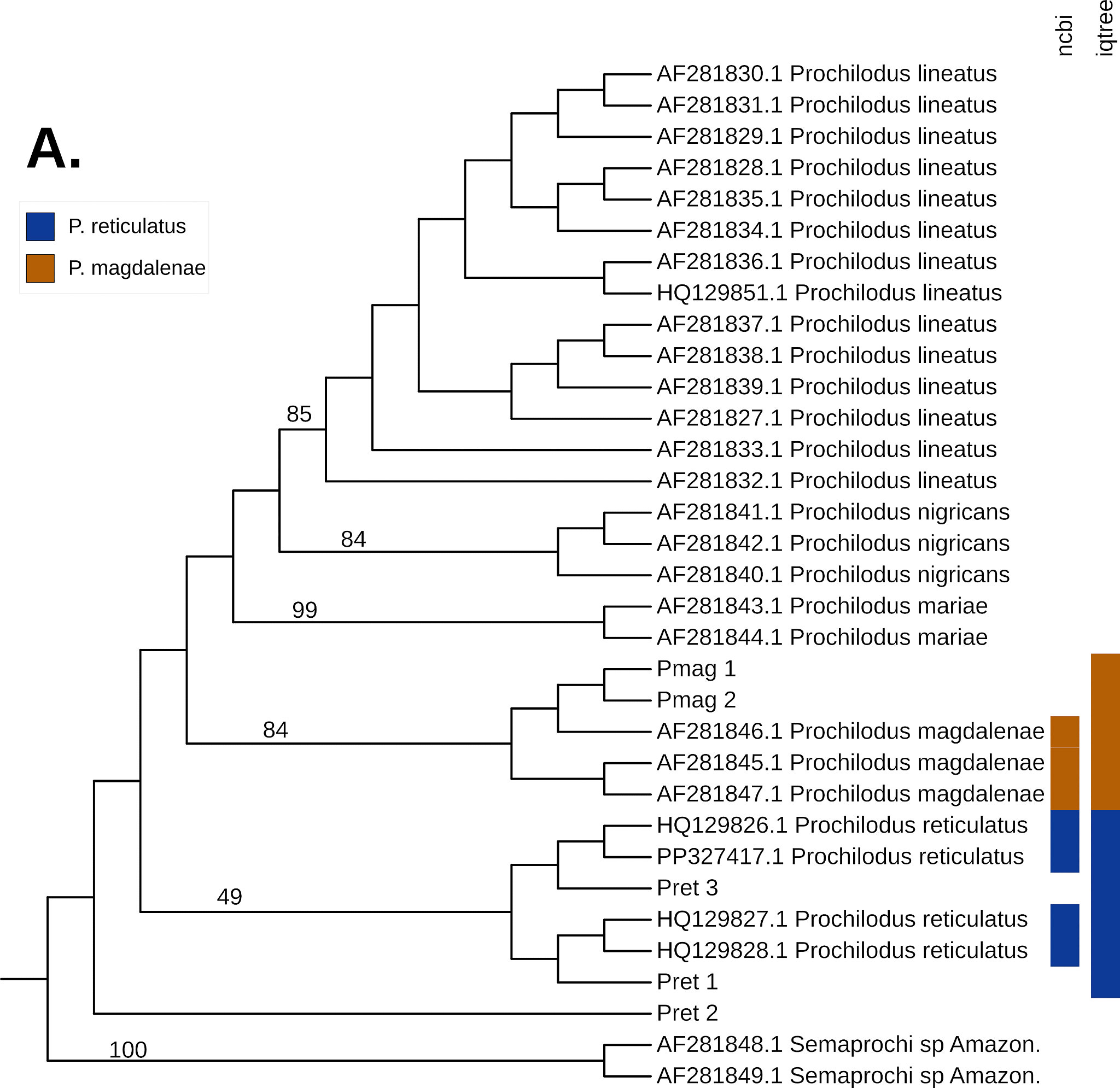

Supplement: Supplementary file 3 [file Image1.jpeg]

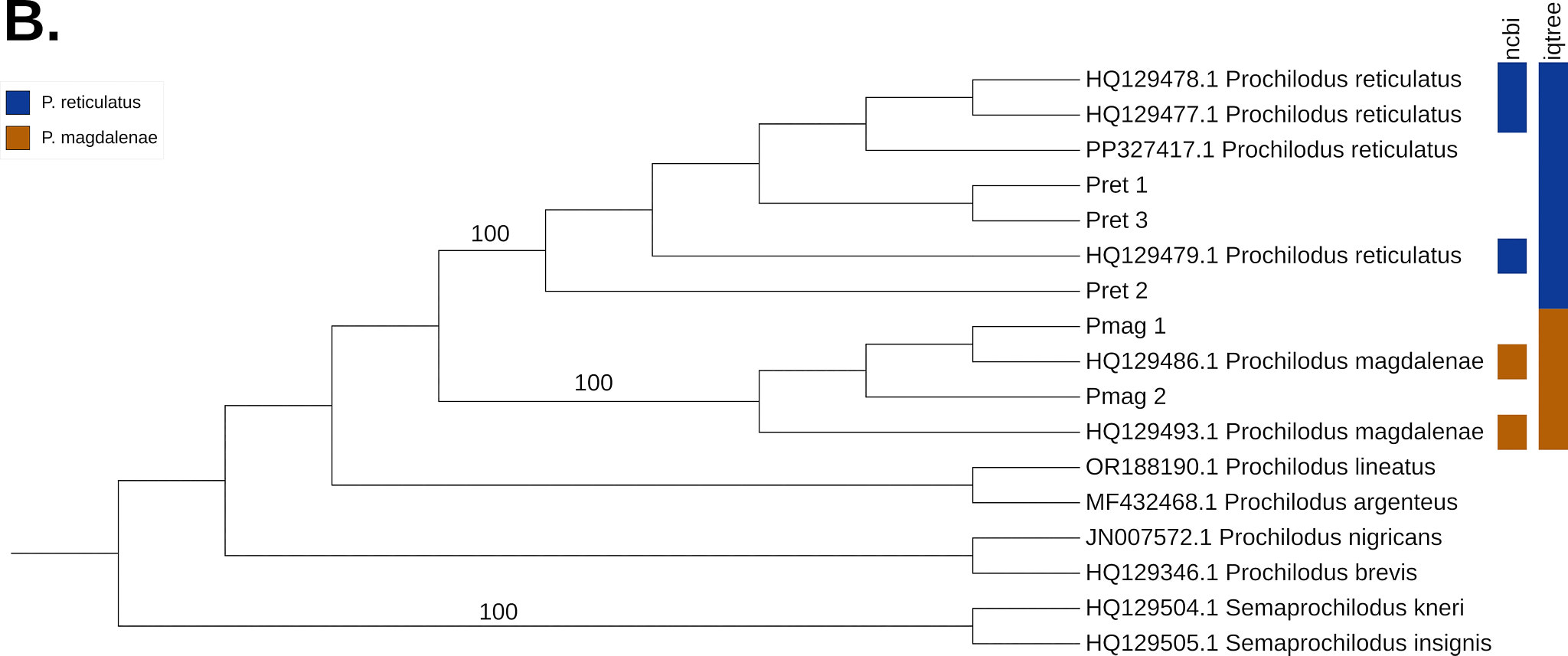

Supplement: Supplementary file 4 [file Image2.jpeg]
